# Excellent patient-reported long-term quality of life after an Ivor Lewis esophagectomy for cancer

**DOI:** 10.3389/fsurg.2025.1491498

**Published:** 2025-07-02

**Authors:** Alisa N. Blumenthaler, Cyrus A. Feizpour, Shadia I. Jalal, Kevin J. Lopez, Neal K. Ramchandani, Joshua McAlister, Susan M. Perkins, Yan Han, Karen M. Rieger, JoAnn Brooks, Kenneth A. Kesler

**Affiliations:** ^1^ Department of Surgery, General Surgery Division, Indiana University Melvin and Bren Simon Cancer Center, Indianapolis, IN, United States; ^2^ Department of Medicine, Medical Oncology Division, Indiana University Melvin and Bren Simon Cancer Center, Indianapolis, IN, United States; ^3^ Department of Radiology, Indiana University Melvin and Bren Simon Cancer Center, Indianapolis, IN, United States; ^4^ Department of Biostatistics and Health Data Science, Indiana University Melvin and Bren Simon Cancer Center, Indianapolis, IN, United States; ^5^ Department of Surgery, Cardiothoracic Surgery Division, Indiana University Melvin and Bren Simon Cancer Center, Indianapolis, IN, United States

**Keywords:** esophageal surgery, esophageal cancer, quality of life, long term survivors, outcomes assessment

## Abstract

**Background:**

Long-term health-related quality of life (HRQOL) may be impacted by upper gastrointestinal tract dysfunction following esophagectomy in up to two-thirds of patients. After esophagectomy, the stomach conduit is passively relying on gravity for drainage. Any resistance to flow through the stomach conduit, therefore, has the potential to significantly impact long-term HRQOL. We have previously reported a side-to-side esophagogastric anastomotic technique, which optimizes anastomotic diameter and vascularity, resulting in a low incidence of leaks and strictures. A wide pyloroplasty is another component of this technique that minimizes resistance to flow. In this study, we aimed to evaluate the long-term HRQOL and esophageal-specific QOL in cancer patients who underwent this surgical approach.

**Methods:**

From 2009–2015, 245 consecutive patients underwent Ivor Lewis esophagectomy for cancer utilizing a consistently performed technique including esophagogastric anastomosis, conduit construction, and surgical pyloric drainage. Functional Assessment of Cancer Therapy-Esophageal (FACT-E) questionnaires were distributed to surviving patients. Routine postoperative fluoroscopic contrast studies were used to characterize conduit function as normal or delayed emptying. Summary statistics for FACT-E and subscales were analyzed.

**Results:**

Eighty-five (34.7%) patients were alive at a median of 58 months and 66 (77.6%) of these patients participated in the study. Survey participants had higher Charlson-Comorbidity Indices (*p* = 0.01) and pathologic tumor stages (*p* = 0.04) compared to non-participants. Participants reported overall very favorable symptom profiles on the Esophageal Cancer Subscale (median, IQR: 55, 48.9–62.0; total possible: 68.0). Early satiety was the only item with >33% negative responses (*n* = 30, 45%). FACT-E total scores were also favorable (median, IQR: 146.0, 126.0–161.0; total possible: 176.0). Delayed emptying, identified in 17 (27.4%) participants, was not associated with HRQOL scores.

**Conclusion:**

Patient-reported long-term HRQOL following a consistently performed esophagectomy technique was very favorable in all subscales, but symptoms of early satiety persisted. Delayed postoperative conduit emptying did not impact HRQOL. These results should be compared to other consistently performed esophagectomy techniques.

## 1 Introduction

Esophagectomy is an important component in the treatment of esophageal cancer but significantly disrupts upper gastrointestinal anatomy (1, 2). As many as two-thirds of patients can experience gastrointestinal dysfunction with detrimental impact on health-related quality of life (HRQOL) (2–6). Postoperative complications may also adversely affect long-term QOL (7–9). Poor postoperative QOL has additionally been identified as a predictor of late mortality (10–12).

While perioperative QOL has previously been studied, long-term HRQOL has primarily been reported after a variety of esophagectomy techniques making interpretation of outcome data challenging (2). The stomach conduit typically utilized for reconstruction is passively relying on gravity for drainage. Any resistance to flow through the stomach conduit, therefore, has potential to significantly impact long-term HRQOL. We have previously described a novel esophagogastric anastomotic technique associated with low leak and stricture rates, which also includes wide surgical pyloric drainage (13). In this study, we aimed to evaluate the long-term HRQOL and esophageal-specific QOL in consecutive cancer patients who underwent this consistently performed surgical approach, including the impact of demographic and oncologic factors, postoperative complications, and conduit function.

## 2 Methods

### 2.1 Study population

After institutional review board approval (protocol #1802249641; 5/18/2018), an institutional database query identified consecutive patients who underwent a previously described Ivor Lewis esophagectomy performed for cancer from 2009 through 2015 (13). In brief, this approach is designed to achieve a stomach conduit without proximal anastomotic or distal pyloric obstruction, including a side-to-side semi-stapled intrathoracic esophagogastric anastomosis which optimizes anastomotic site vascularity and diameter. This technique also involves a consistent approach to conduit construction and orientation, with wide pyloric drainage defined by a longitudinal pyloric incision which allows passage of an index finger proximally and distally before transverse two-layer suture closure (14). A generous Kocher maneuver allows a well-vascularized stomach conduit to be advanced at or above the azygous arch for anastomosis while straightening the conduit route directly into the small bowel. Minimizing proximal esophageal dissection is also important to avoid devascularization.

### 2.2 Patient data

Data collected included demographic data, cancer and treatment-related variables, preoperative comorbidities, and postoperative complications. Charlson-Comorbidity Index scores were calculated and Clavien-Dindo postoperative complication grades (scale 0–5) were recorded (15, 16). Routine fluoroscopic gastrografin upper gastrointestinal studies performed between postoperative days 5–7 to assess for anastomotic leak were retrospectively reviewed by an expert radiologist (JM) who was blinded to QOL outcomes. Gastric conduit function was classified based on the radiologist's report as normal or minimal emptying delay (NE = normal emptying) and moderate to severe emptying delay (DE = delayed emptying). Although the time of contrast passage was not measured, it is estimated that contrast transit time into the small bowel was less than one minute in patients with NE, and greater than one minute in patients with DE (Supplementary Figure S1). Patients' vital statuses were updated utilizing an institutional database.

### 2.3 HRQOL survey administration and analysis

Surviving patients were contacted to complete Functional Assessment of Cancer Therapy–Esophageal (FACT-E) questionnaires (17). Patients were offered a $50 (USD) gift certificate for participation. The FACT-E includes the 27 standard items in the Functional Assessment of Cancer Therapy–General (FACT-G) and 17 questions regarding symptoms specific to patients with esophageal cancer [esophageal cancer subscale (ECS)]. Patients grade their symptoms on a 5-point Likert scale from “not at all” (0) to “very much” (4). Scoring of negatively worded questions was reversed so that a higher point value indicates a better QOL. Sum scores were calculated for the FACT-E total score, the FACT-G total score, the physical well-being (PWB), social well-being (SWB), emotional well-being (EWB), functional well-being (FWB), and the ECS subscales. The swallowing index includes 5 ECS items related to general ease of swallowing, difficulty with swallowing liquids, semi-solid and solid foods, and choking when swallowing. The eating subscale consists of 3 ECS items related to the ability to eat the kinds and volumes of food desired, and enjoyment of eating.

### 2.4 Statistical analysis

Categorical variables were summarized by frequency and percentage, and continuous variables were summarized by mean and standard deviation, or median and interquartile range (IQR). Comparisons between groups were performed using chi-square tests or Fisher's exacts test for categorical variables, and two-sample *t*-tests or Wilcoxon rank-sum tests for continuous variables. Overall survival times were calculated from the date of surgery to the date of survey request or date of death. The median and 95% confidence interval (CI) of follow-up time and overall survival time were estimated from Kaplan–Meier analyses.

Univariate analyses of the QOL data were performed utilizing Spearman's correlation or Wilcoxon rank-sum tests, due to non-normality of the QOL data. Variables which had predictive *p*-value < 0.20 by univariate analysis were included in the multivariable linear regressions. Box-Cox transformations were conducted for the outcomes in which residuals were not normally distributed. Analyses were performed using SAS v9.4 (Cary, NC). Missing data was handled in accordance with the guidelines provided with the FACT-E instrument (18, 19). There was little missing data for all descriptive and univariable analyses and missing values were ignored. For the multivariable analysis, a complete-case analysis was used. The level of statistical significance was set at *α* = 0.05.

## 3 Results

### 3.1 Patient characteristics: survivors vs. non-survivors

Eighty-five of 245 identified patients (34.7%) were alive at the time of survey distribution after a median follow-up of 58 months (95% CI: 49.8–63.6 months). The median survival for non-survivors was 16.4 months (95% CI: 12.1–19.8). Non-survivors had more cardiac comorbidities, more advanced tumor stages, higher Clavien-Dindo classifications, more pulmonary complications, and more frequently received neoadjuvant therapy compared to survivors (Supplementary Table S1). There were no other differences between the two groups including rates of anastomotic leak (survivors *n* = 2, 2.4% vs. non-survivors *n* = 4, 2.5%, *p* = 1.00) or subsequent need for anastomotic dilation (survivors *n* = 5, 5.9% vs. non-survivors *n* = 6, 3.8%, *p* = 0.52). No patient required pyloric intervention postoperatively.

### 3.2 Patient characteristics: participants vs. non-participants

The demographic and clinical data of survey participants (*n* = 66, 77.6%) and non-participants (*n* = 19, 22.4%) are shown in Table 1. Participants were older, with higher Charlson-Comorbidity Indices, and higher pathologic tumor stages. The two groups had similar rates of postoperative morbidity except for anastomotic leaks (*n* = 2, 10.5% non-participants vs. *n* = 0, 0.0% participants, *p* = 0.048) and need for anastomotic dilation (*n* = 4, 21.1% non-participants vs. *n* = 1, 1.5% participants, *p* < 0.01). Overall, 75% of patients demonstrated NE on postoperative contrast studies, while 16.7% and 27.4% of non-participants and participants had DE, respectively (*p* = 0.54). At last follow-up, 7.6% of participants had known disease recurrence, compared to 10.5% of non-participants (*p* = 0.65).

**Table 1:** Characteristics of survey participants and non-participants.

Variable	Overall	Non-participants	Participants	*P*-value^a^
	*N* = 85	*N* = 19	*N* = 66	
Age at surgery, mean (SD)	60.46 ± 10.33	53.72 ± 11.84	62.40 ± 9.06	0.001
Female, *n* (%)	11 (12.94%)	3 (15.79%)	8 (12.12%)	0.70
Charlson Comorbidity Index, median (IQR)	4.0 (3.0, 5.0)	3.0 (2.0, 5.0)	4.0 (3.0, 5.0)	0.01
Cardiac comorbidity, *n* (%)	13 (15.29%)	4 (21.05%)	9 (13.64%)	0.48
Diabetes Mellitus, *n* (%)	19 (22.35%)	3 (15.79%)	16 (24.24%)	0.54
COPD, *n* (%)	6 (7.06%)	1 (5.26%)	5 (7.58%)	1.00
Histology, *n* (%)				0.54
-Adenocarcinoma	82 (96.47%)	18 (94.74%)	64 (96.97%)	
-Squamous	3 (3.53%)	1 (5.26%)	2 (3.03%)	
Clinical TNM stage, *n* (%)^b^				0.81
-0	2 (2.47%)	0 (0.0%)	2 (3.17%)	
-I	16 (19.75%)	2 (11.11%)	14 (22.22%)	
-II	17 (20.99%)	4 (22.22%)	13 (20.63%)	
-III	36 (44.44%)	9 (50.00%)	27 (42.86%)	
-IV	10 (12.35%)	3 (16.67%)	7 (11.11%)	
Neoadjuvant Therapy, *n* (%)	61 (71.76%)	15 (78.95%)	46 (69.70%)	0.43
Tumor location, *n* (%)^b^				0.54
-Upper	1 (1.19%)	0 (0.0%)	1 (1.54%)	
-Middle	2 (2.38%)	1 (5.26%)	1 (1.54%)	
-Lower	81 (96.43%)	18 (94.74%)	63 (96.92%)	
Pathologic TNM stage, *n* (%)^b^				0.04
-0	8 (9.52%)	1 (5.26%)	7 (10.77%)	
-I	52 (61.90%)	12 (63.16%)	40 (61.54%)	
-II	8 (9.52%)	5 (26.32%)	3 (4.62%)	
-III	15 (17.86%)	1 (5.26%)	14 (21.54%)	
-IV	1 (1.19%)	0 (0.0%)	1 (1.54%)	
Clavien Dindo Classification, *n* (%)				0.40
-0	54 (63.53%)	11 (57.89%)	43 (65.15%)	
-1	5 (5.88%)	2 (10.53%)	3 (4.55%)	
-2	18 (21.18%)	3 (15.79%)	15 (22.73%)	
-3	4 (4.71%)	1 (5.26%)	3 (4.55%)	
-4	4 (4.71%)	2 (10.53%)	2 (3.03%)	
Pulmonary Complications, *n* (%)	13 (15.29%)	4 (21.05%)	9 (13.64%)	0.48
Atrial Arrythmias, *n* (%)	15 (17.65%)	3 (15.79%)	12 (18.18%)	1.00
Other Complications, *n* (%)	13 (15.29%)	5 (26.32%)	8 (12.12%)	0.15
Postoperative Conduit Function, *n* (%)^b^				0.54
-Normal Emptying	60 (75.00%)	15 (83.33%)	45 (72.58%)	
-Delayed Emptying	20 (25.00%)	3(16.67%)	17(27.42%)	
Disease Recurrence, *n* (%)	7(8.24%)	2(10.53%)	5(7.58%)	0.65

^a^ From chi-square test or Fisher's exact test for categorical variables, two-sample *t*-test for age, and Wilcoxon rank-sum test for Charlson comorbidity index.

^b^ Missing data: Clinical TNM stage *n* = 4; Tumor location *n* = 1; Pathologic TNM stage *n* = 1; Postoperative Conduit Function *n* = 5.

### 3.3 Overall patient-reported HRQOL and esophageal-specific QOL

**Figure 1:**
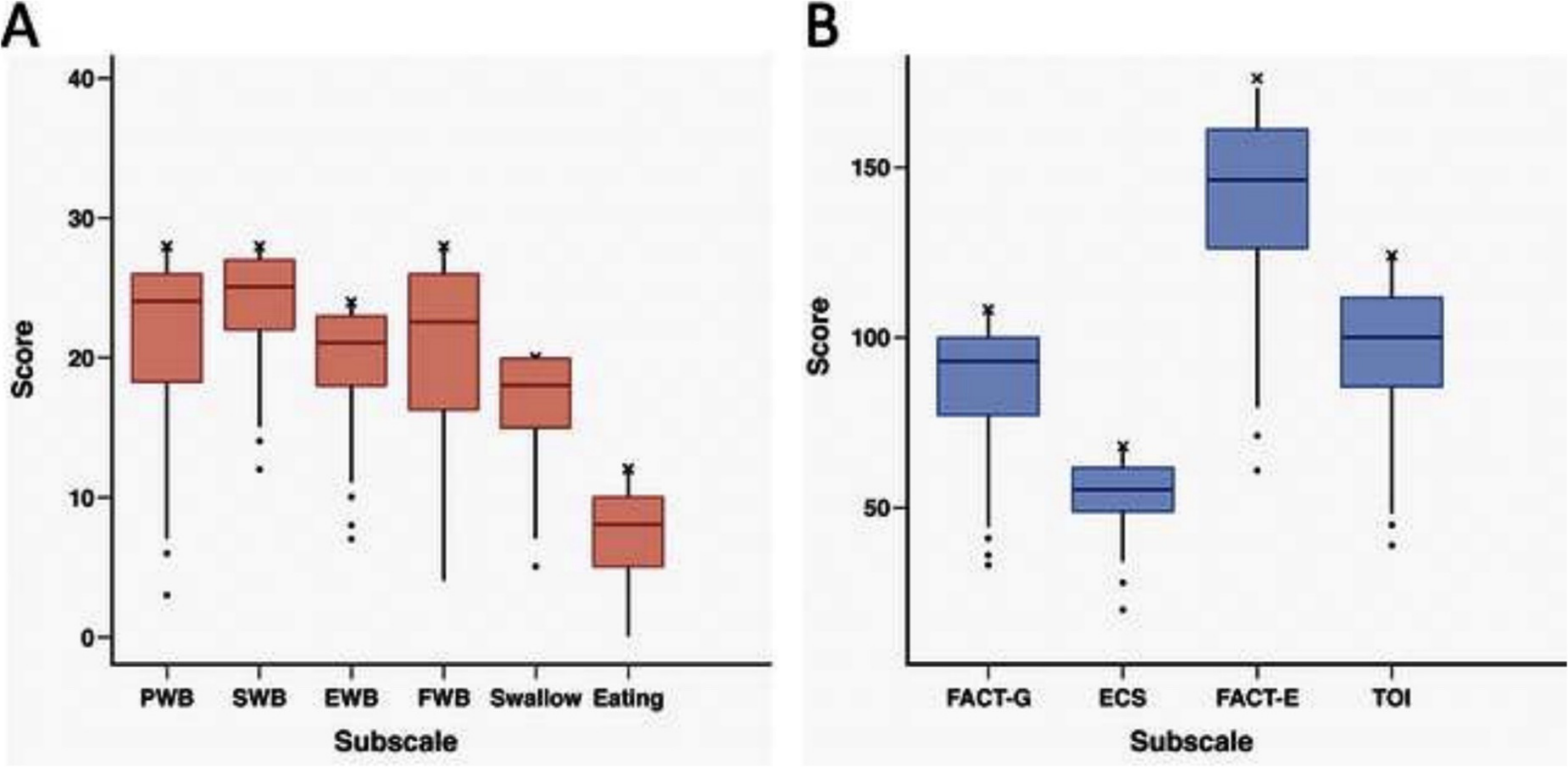
**(A,B)** Box and whisker plots for FACT-E subscale distribution. Upper and lower borders of box represent 25th and 75th percentiles, middle horizontal line represents the median, upper and lower whiskers represent the minimum and maximum values of non-outliers. Outliers are included as extra dots lying outside the whiskers. “X” indicates the maximum possible score for each subscale. PWB, physical well-being; SWB, social well-being; EWB, emotional well-being; FWB, functional well-being; FACT-G, functional assessment cancer treatment–General; ECS, esophageal cancer subscale; TOI, trial outcome index. *n* = 66, except for EWB, Swallow, FACT-G, and FACT-E where *n* = 65.

A majority of patients reported minimal symptomatology on FACT-G and ECS items (Tables 2, 3). Early satiety was the only adverse ECS item for which greater than one third (45%) of patients reported. Only 26% reported “quite a bit” or “very much” in response to being “able to eat as much food as I want”. Forty-eight patients (74% of responders) reported being able to swallow naturally and easily “quite a bit” or “very much”, with a minority of patients reporting difficulty swallowing solid foods (*N* = 8, 12%), soft foods (*N* = 2, 3%), or liquids (*N* = 0, 0%). One patient (2%) reported choking with swallowing “quite a bit” or “very much”. While symptoms of dumping syndrome are not included in the FACT-E survey, 10 patients (15%) experienced significant abdominal pain which could represent a surrogate symptom, though none reported weight loss. Figure 1 depicts the distribution of scores on the FACT-E subscales. FACT-E total scores were favorable (median, IQR: 146.0, 126.0–161.0; total possible: 176.0). The median ECS score was 55 (48.9–62.0, total possible: 68). Three survey responders were outliers in multiple subscales, while five others were outliers in a single subscale. No obvious trends were identified in outliers, as most had earlier stage disease, experienced only minor or no postoperative complications and demonstrated NE.

**Table 2:** Patients' responses to esophageal cancer subscale (ECS) items of the functional assessment of cancer therapy–esophageal (FACT-E) survey. Highlighted cells signify responses that correlate with better quality of life.

Survey question	Not at all/A little bit	Somewhat	Quite a bit/Very Much
I am able to eat the foods that I like…	10 (15%)	17 (26%)	39 (59%)
My mouth is dry…^a^	43 (66%)	15 (23%)	7 (11%)
I have trouble breathing…	46 (70%)	13 (20%)	7 (11%)
My voice has its usual quality and strength…^a^	6 (9%)	9 (14%)	50 (77%)
I am able to eat as much food as I want…	30 (45%)	19 (29%)	17 (26%)
I am able to communicate with others…	1 (2%)	2 (3%)	63 (95%)
I can swallow naturally and easily…^a^	8 (12%)	9 (14%)	48 (74%)
I have difficulty swallowing solid foods…^a^	49 (75%)	8 (12%)	8 (12%)
I have difficulty swallowing soft or mashed foods…	60 (91%)	4 (6%)	2 (3%)
I have difficulty swallowing liquids…^a^	62 (95%)	3 (5%)	0 (0%)
I have pain in my chest when I swallow…^a^	62 (95%)	3 (5%)	0 (0%)
I choke when I swallow…^c^	55 (87%)	7 (11%)	1 (2%)
I am able to enjoy meals with family or friends…	11 (17%)	10 (15%)	45 (68%)
I wake at night because of coughing…^b^	51 (80%)	7 (11%)	6 (9%)
I have a good appetite…	12 (18%)	12 (18%)	42 (64%)
I have pain in my stomach area…	45 (68%)	11 (17%)	10 (15%)
I am losing weight…^a^	54 (83%)	6 (9%)	5 (8%)

Missing data: ^a^*n* = 1; ^b^*n* = 2; ^c^*n* = 3.

**Table 3:** Patients' responses to functional assessment of cancer therapy–general (FACT-G) questionnaire items. Highlighted cells signify responses that correlate with better quality of life.

Survey question	Not at all/A little bit	Somewhat	Quite a bit/Very Much
I have a lack of energy^a^	38 (58%)	10 (15%)	17 (26%)
I have nausea	50 (76%)	6 (9%)	10 (15%)
Because of my physical condition, I have trouble meeting the needs of my family	53 (80%)	6 (9%)	7 (11%)
I have pain^a^	44 (68%)	9 (14%)	12 (18%)
I am bothered by side effects of treatment^b^	41 (64%)	12 (19%)	11 (17%)
I feel ill	56 (85%)	4 (6%)	6 (9%)
I am forced to spend time in bed	60 (91%)	4 (6%)	2 (3%)
I feel close to my friends	7 (11%)	7 (11%)	52 (79%)
I get emotional support from my family^a^	0 (0%)	1 (2%)	64 (98%)
I get support from my friends^a^	3 (5%)	12 (18%)	50 (77%)
My family has accepted my illness	0 (0%)	2 (3%)	64 (97%)
I am satisfied with family communication about my illness	0 (0%)	4 (6%)	62 (94%)
I feel close to my partner (or the person who is my main support)^b^	1 (2%)	2 (3%)	61 (95%)
I am satisfied with my sex life^c^	19 (43%)	9 (20%)	16 (36%)
I feel sad^a^	55 (85%)	7 (11%)	3 (5%)
I am satisfied with how I am coping with my illness^a^	7 (11%)	12 (18%)	46 (71%)
I am losing hope in the fight against my illness^a^	59 (91%)	5 (8%)	1 (2%)
I feel nervous^a^	53 (82%)	8 (12%)	4 (6%)
I worry about dying^a^	58 (89%)	4 (6%)	3 (5%)
I worry that my condition will get worse^a^	48 (74%)	9 (14%)	8 (12%)
I am able to work (include work at home)	15 (23%)	9 (14%)	44 (64%)
My work (include work at home) is fulfilling^a^	9 (14%)	10 (15%)	46 (71%)
I am able to enjoy life	6 (9%)	11 (17%)	49 (74%)
I have accepted my illness	2 (3%)	8 (12%)	56 (85%)
I am sleeping well	12 (18%)	18 (27%)	36 (55%)
I am enjoying the things I usually do for fun^a^	13 (20%)	6 (9%)	46 (71%)
I am content with the quality of my life right now^a^	11 (17%)	8 (12%)	46 (71%)

Missing data: ^a^*n* = 1; ^b^*n* = 2; ^c^*n* = 22.

### 3.4 Associations to patient-reported HRQOL and esophageal-specific QOL

On univariate analysis, few patient characteristics were associated with HRQOL subscale scores (Supplementary Table S2). Increasing age was positively correlated with the swallowing subscale score (*r*_s_ = 0.28, *p* = 0.02). Postoperative complications (Clavien-Dindo grades 2–4 vs. grades 0–1) were associated with lower SWB (*p* = 0.04) and a trend toward lower FWB (*p* = 0.08) and FACT-E total scores (*p* = 0.09). There was no correlation between postoperative conduit emptying grade and subscale scores. Supplementary Table S3 shows the results of multivariable linear regression analysis. Multivariable analysis indicates that increasing age was associated with better QOL for EWB (*p* = 0.01), Swallowing (*p* = 0.04), and FACT-E Total Score (*p* < 0.01). Better Swallowing scores were identified in females (*p* = 0.01) and patients treated with neoadjuvant therapy (*p* = 0.01). Patients with clinical stage III-IV (vs. stage 0-II, *p* < 0.01) and patients with disease recurrence (*p* = 0.03) had worse Swallowing scores.

## 4 Discussion

Assessing long-term HRQOL and esophageal-specific QOL after esophagectomy is essential to understand the impact of treatment in cancer patients. Comparing outcomes between esophagectomy techniques and identifying patients at risk of poor QOL outcomes is important in this regard. Previous reports have demonstrated a significant decline in HRQOL immediately following surgery, with gradual improvement before reaching a plateau which is often not equivalent to preoperative status (3, 19–21). Specific symptoms, such as reflux, eating difficulties, loss of appetite, and diarrhea can impact QOL for up to ten years following surgery (1). After this esophagectomy approach, a majority of patients in our cohort reported very favorable long-term HRQOL and esophageal-specific QOL outcomes.

Emptying of the primarily passive stomach conduit, promoted largely by gravity, can be optimized by the creation of a narrow but adequately sized conduit with a uniform diameter and no redundancy (22). One may speculate that creation of a gastric conduit with a relatively narrow diameter promotes good conduit emptying at the “price” of early satiety which is common after esophagectomy; 45% of our cohort responded negatively to “I am able to eat as much as I want”, consistent with prior studies (2, 3, 5, 6, 23) Deldycke evaluated 322 patients who underwent an Ivor Lewis approach utilizing a whole stomach conduit and a routine pyloroplasty and found a 60% incidence of early satiety up to 5 years after surgery (6). While surgical pyloric drainage may promote conduit function, it may also promote dumping. Boshier reported an 87% incidence of early satiety in 171 patients at least 3 months post-esophagectomy (2). Inclusion of intraoperative pyloric drainage or postoperative pyloric dilation was associated with a decreased incidence of early satiety but higher rates of dumping symptoms. Although early satiety was the most common symptom in our study, the incidence was notably lower than the 87% and 60% reported by Boshier and Deldycke. None of the patients in our series required postoperative pyloric dilation, suggesting that surgical pyloric drainage can be beneficial to conduit function.

A unique aim of our study was to explore the relationship between postoperative fluoroscopic conduit function and long-term symptomatology. Clinically relevant DE occurs in 10%–20% of patients and may be affected by surgical technique (24). Using our technique, DE in the immediate postoperative period did not appear to impact long-term HRQOL. We speculate that DE identified in 27% of our series, despite wide surgical pyloric drainage, may be attributable to temporary conditions such as pyloric swelling or narcotic use. It is also possible that minor emptying delay if chronic, does not significantly impact perceived QOL. Dedicated prospective study of long-term QOL outcomes with respect to conduit function after various esophagectomy techniques would be of interest.

Long-term HRQOL in our study compares favorably to findings reported by Gutschow, with approximately half of their 147 patient cohort reporting QOL similar to a healthy reference population after Ivor Lewis esophagectomy (25). The patients in our study reported mean scores on the FACT-G and subscales similar to, and in some cases, higher than those previously reported in a healthy population (26). The consistent surgical approach in the current series may explain the concentration of scores within a narrow range on QOL subscales. There were, however, a distinct minority of outlier patients reporting poor QOL in multiple or isolated subscales without obvious trends. Further investigation is needed to identify patients with the potential to be outliers with respect to HRQOL and efforts made to minimize these outcomes.

Anastomotic technique is an important consideration as anastomotic complications can impact long-term symptomatic outcomes. Reported rates of anastomotic leak and stricture after end-to-end circular anastomotic stapling averages 15% and 17% respectively (14, 27, 28). In comparison, we have previously reported leak and stricture rates which are three- to four-fold lower, 4% and 5% respectively, in patients undergoing the side-to-side anastomosis technique utilized in the current study (14). Significantly lower leak rates after linear stapled as compared to end-to-end circular stapling anastomotic techniques are consistent with several other reports (29–31). While mortality from anastomotic leaks may be decreasing, strictures occur in up to 56% of anastomotic leaks and can influence long-term symptoms (21, 28). Low rates of dysphagia and choking with swallowing in our series are arguably reflective of a technique that optimizes long-term function by minimizing anastomotic narrowing or stricture.

While our technique has remained consistent over time, we have recently introduced minor modifications. First, an approximate 45-degree angled wedge is removed from the lesser curve conduit tip using a double row GIA stapler, creating a more vascularized three to four cm “platform” for the future site of the intrathoracic anastomosis. We have also been closing the open end of the triangulated anastomosis with two to five sequential fires of an endo GIA tristapler, utilizing staples on only one side of the stapler knife to minimize the amount of tissue resected, then oversewing the staple line. Both are relatively minor modifications intended to further reduce anastomotic leaks. Since our publication in 2018, we have additionally been utilizing cryotherapy to the interspace entry site and two intercostal nerves above and below which has significantly improved postoperative analgesia and recovery after thoracotomy. Although there is a trend towards minimally invasive esophagectomy approaches as short-term QOL outcomes appear to be superior, we believe that intercostal cryotherapy has greatly mitigated any increased pain issues related to this open approach.

Existing literature fails to agree regarding the impact of postoperative complications on QOL (8, 9, 20). The LASER study found that the absence of 30-day postoperative complications was associated with improved physical and social functioning after 4.3 years (23). Alternatively, a prospective cohort study by Jezerskyte identified no overall QOL differences comparing patients with and without complications up to 24 months following surgery (20). While our univariate analysis identified an association of grade 2 or higher complications with lower scores in multiple FACT-E subscales, this did not remain significant by multivariable analysis. Rather, more advanced clinical tumor stage and the presence of disease recurrence were associated with lower scores on the Swallowing subscale. Increasing age was associated with better scores in multiple domains which may reflect the subjectivity of QOL and how perception of symptoms can change with age. Further study in this regard would be of interest.

Our study has limitations. As we measured QOL in long-term survivors, a selection bias for patients with lower mortality risk and, thus, better QOL is possible. While possible biases may exist between participants and non-participants, such as differences in anastomotic leak or stricture, the absolute numbers of these complications were small and, therefore, unlikely to influence overall outcomes. Patients surviving longer may establish a “new normal”, subjectively perceiving fewer symptoms over time, an evolution of symptoms which is not captured by our cross-sectional study (32).

As advances in multimodality therapy and immunotherapy continue to improve the prognoses of esophageal cancer patients, optimizing long-term QOL becomes increasingly important. Esophagectomy techniques which minimize complications and promote conduit function likely hold promise to improve long-term esophageal-specific QOL. In this series, our surgical approach to esophagectomy led to very favorable patient-reported long-term QOL which arguably can be attributed to the good function of a passive conduit. Future studies evaluating long-term QOL would benefit from a focus on other consistently performed surgical approaches for comparison.

## Data Availability

The raw data supporting the conclusions of this article will be made available by the authors, without undue reservation.
