# Supplementary material for: Excellent patient-reported long-term quality of life after an Ivor Lewis esophagectomy for cancer
Source: Front Surg. 2025 Jul 2;12:1491498. doi: 10.3389/fsurg.2025.1491498 (PMC12263660; doi:10.3389/fsurg.2025.1491498)

Supplemental Data and Figures

Supplemental Table 1. Demographic, clinical, oncologic, and perioperative characteristics of patients who survived and those who did not.

|  |  | **Vital Status** | |  |
| --- | --- | --- | --- | --- |
| **Variable** | **Overall N=245** | **Survivors N=85** | **Non-survivors N=160** | **P-value^1^** |
|  |  |  |  |  |
| Age at surgery, mean ± SD | 61.56 ± 10.43 | 60.46 ± 10.33 | 62.15 ± 10.46 | 0.229 |
| Female Gender, n(%) | 32 (13.06%) | 11 (12.94%) | 21 (13.13%) | 0.968 |
| Charlson Comorbidity Index,  median (IQR) | 4.0 (3.0, 5.0) | 4.0 (3.0, 5.0) | 4.0 (3.0, 6.0) | 0.015 |
| Cardiac comorbidity, n(%) | 69 (28.16%) | 13 (15.29%) | 56 (35.00%) | 0.001 |
| Diabetes Mellitus, n(%) | 63 (26.47%) | 19 (22.35%) | 44 (28.76%) | 0.283 |
| COPD, n(%) | 28 (12.12%) | 6 (7.06%) | 22 (15.07%) | 0.072 |
| Histology, n(%) |  |  |  | 0.421 |
| - Adenocarcinoma | 239 (97.55%) | 82 (96.47%) | 157 (98.13%) |  |
| - Squamous | 6 (2.45%) | 3 (3.53%) | 3 (1.88%) |  |
| Clinical TNM Stage, n(%) |  |  |  | 0.002 |
| - 0 | 3 (1.26%) | 2 (2.47%) | 1 (0.64%) |  |
| - I | 25 (10.50%) | 16 (19.75%) | 9 (5.73%) |  |
| - II | 39 (16.39%) | 17 (20.99%) | 22 (14.01%) |  |
| - III | 135 (56.72%) | 36 (44.44%) | 99 (63.06%) |  |
| - IV | 36 (15.13%) | 10 (12.35%) | 26 (16.56%) |  |
| Neoadjuvant Therapy, n(%) | 200 (81.63%) | 61 (71.76%) | 139 (86.88%) | 0.004 |
| Tumor location, n(%) |  |  |  | 1.000 |
| - Upper | 2 (0.83%) | 1 (1.19%) | 1 (0.63%) |  |
| - Middle | 6 (2.48%) | 2 (2.38%) | 4 (2.53%) |  |
| - Lower | 234 (96.69%) | 81 (97.59%) | 81 (96.43%) |  |
| Pathologic TNM Stage, n(%) |  |  |  | <0.001 |
| - 0 | 10 (4.10%) | 8 (9.52%) | 2 (1.25%) |  |
| - I | 104 (42.62%) | 52 (61.90%) | 52 (32.50%) |  |
| - II | 29 (11.89%) | 8 (9.52%) | 21 (13.13%) |  |
| - III | 75 (30.74%) | 15 (17.86%) | 60 (37.50%) |  |
| - IV | 26 (10.66%) | 1 (1.19%) | 25 (15.63%) |  |
| Clavien-Dindo Classification, n(%) |  |  |  | 0.003 |
| - 0 | 117 (47.76%) | 54 (63.53%) | 63 (39.38%) |  |
| - 1 | 19 (7.76%) | 5 (5.88%) | 14 (8.75%) |  |
| - 2 | 55 (22.45%) | 18 (21.18%) | 37 (23.13%) |  |
| - 3 | 18 (7.35%) | 4 (4.71%) | 14 (8.75%) |  |
| - 4 | 33 (13.47%) | 4 (4.71%) | 29 (18.13%) |  |
| - 5 | 3 (1.22%) | 0 (0.0%) | 3 (1.88%) |  |
| Pulmonary Complications, n(%) | 79 (32.24%) | 13 (15.29%) | 66 (41.25%) | <0.001 |
| Atrial Arrythmias, n(%) | 54 (22.04%) | 15 (17.65%) | 39 (24.38%) | 0.227 |
| Other Complications, n(%) | 48 (19.59%) | 13 (15.29%) | 35 (21.88%) | 0.217 |
| Clinical status, n(%) |  |  |  | -- |
| - Alive with no evidence of disease | 78 (31.84%) | 78 (91.76%) | - |  |
| - Alive with recurrence | 7 (2.86%) | 7 (8.24%) | - |  |
| - Death without recurrence | 93 (37.96%) | - | 93 (58.13%) |  |
| - Death with recurrence | 67 (27.35%) | - | 67 (41.88%) |  |

^1^From chi-square test or Fisher’s Exact test for categorical variables, two-sample t-test for age, and Wilcoxon rank-sum test for Charlson Comorbidity Index.

Supplemental Table 2. Univariate analysis of clinical and pathologic features associated with QOL subscale scores.

| Variable | | r_s_ or median (25^th^ percentile, 75^th^ percentile)  P-value | | | | | | | |
| --- | --- | --- | --- | --- | --- | --- | --- | --- | --- |
|  |  | PWB | SWB | EWB | FWB | ECS | Swallowing | Eating | FACT-E Total |
| Age* | | 0.01  0.96 | 0.05  0.68 | 0.13  0.31 | -0.04  0.76 | 0.19  **0.14** | 0.28  **0.02** | 0.16  **0.20** | 0.13  0.30 |
| Gender^┼^ | Male | 23.7 (18.0, 26.0) | 25.0 (22.0, 28.0) | 21.0 (18.0, 23.0) | 23.0 (16.0, 26.0) | 54.5 (48.9, 62.0) | 18.0 (14.0, 20.0) | 8.0 (5.0, 10.0) | 147.0 (126.0, 162.0) |
|  | Female | 24.5 (17.0, 25.5) | 24.5 (19.1, 25.5) | 16.5 (14.5, 22.5) | 22.0 (17.0, 24.0) | 55.0 (45.5, 60.7) | 20.0 (16.0, 20.0) | 6.50 (3.0, 9.5) | 138.5 (113.0, 157.2) |
|  | p-value | 0.77 | 0.28 | **0.12** | 0.55 | 0.76 | 0.36 | 0.36 | 0.36 |
| Charlson Comorbidity Index* | | -0.09  0.49 | -0.04  0.78 | 0.02  0.89 | -0.13  0.30 | -0.004  0.97 | 0.11  0.41 | 0.03  0.78 | -0.03  0.84 |
| Neoadjuvant Therapy ^┼^ | Yes | 23.0 (18.0, 26.0) | 25.0 (22.2, 28.0) | 21.0 (18.0, 23.0) | 22.0 (14.0, 26.0) | 53.5 (47.0, 62.0) | 18.0 (15.0, 20.0) | 8.0 (4.0, 10.0) | 143.5 (119.7, 161.0) |
|  | No | 24.0 (20.0, 26.0) | 25.0 (21.0, 26.5) | 22.0 (19.0, 24.0) | 24.0 (19.5, 28.0) | 56.5 (50.0, 61.5) | 18.5 (15.0, 20.0) | 8.0 (7.0, 10.0) | 148.0 (139.0, 161.0) |
|  | p-value | 0.69 | 0.47 | **0.10** | 0.21 | 0.54 | 0.97 | 0.58 | 0.39 |
| Clinical TNM Stage ^┼^ | 0, I-II | 24.0 (19.0, 26.0) | 24.0 (23.0, 27.0) | 21.5 (18.5, 24.0) | 24.0 (19.0, 28.0) | 56.0 (50.0, 62.0) | 19.0 (15.0, 20.0) | 8.0 (6.0, 10.0) | 147.8 (124.5, 160.5) |
|  | III-IV | 23.0 (18.0, 26.0) | 25.0 (22.2, 28.0) | 21.0 (18.0, 23.0) | 22.0 (15.0, 26.0) | 55.0 (47.0, 62.0) | 18.0 (15.0, 20.0) | 8.0 (5.0, 10.0) | 143.5 (126.0, 162.0) |
|  | p-value | 0.69 | 0.35 | 0.62 | **0.20** | 0.81 | 0.62 | 0.94 | 0.67 |
| Clavien-Dindo ^┼^ | 0-1 | 24.0 (19.0, 26.0) | 25.4 (23.0, 28.0) | 22.0 (19.0, 23.0) | 24.0 (19.0, 26.0) | 56.0 (49.9, 62.0) | 18.5 (14.0, 20.0) | 8.0 (7.0, 10.0) | 149.5 (133.0, 161.0) |
|  | 2-4 | 21.5 (15.0, 26.0) | 24.0 (21.0, 25.5) | 18.5 (16.0, 22.9) | 19.5 (13.5, 24.0) | 51.0 (45.0, 59.0) | 18.0 (16.0, 20.0) | 5.5 (3.0, 10.0) | 134.4 (110.2, 154.5) |
|  | p-value | 0.38 | **0.04** | **0.11** | **0.08** | 0.27 | 0.63 | 0.22 | **0.09** |
| Conduit Emptying Grade ^┼^ | NE | 23.0 (18.0, 26.0) | 25.0 (23.0, 27.0) | 21.0 (18.0, 23.5) | 22.0 (15.0, 26.0) | 56.0 (48.0, 62.0) | 18.0 (16.0, 20.0) | 8.0 (5.0, 10.0) | 145.0 (122.5, 161.5) |
|  | DE | 24.0 (20.0, 26.0) | 25.0 (20.0, 27.0) | 19.0 (18.0, 23.0) | 23.0 (16.0, 26.0) | 52.0 (49.9, 59.0) | 17.0 (14.0, 19.5) | 8.0 (4.0, 10.0) | 147.0 (127.0, 156.0) |
|  | p-value | 0.54 | 0.92 | 0.62 | 0.76 | 0.52 | 0.54 | 0.87 | 0.80 |
| Pathologic TNM stage ^┼^ | 0, I-II | 23.5 (18.0, 26.0) | 25.0 (23.0, 27.0) | 21.0 (18.0, 24.0) | 23.0 (15.0, 26.0) | 52.5 (47.0, 62.0) | 18.0 (15.0, 20.0) | 8.0 (5.0, 10.0) | 146.0 (121.0, 162.0) |
|  | III-IV | 25.0 (21.0, 26.0) | 25.0 (21.0, 27.0) | 21.5 (18.0, 23.0) | 22.0 (17.0, 26.0) | 57.0 (50.0, 61.0) | 19.0 (14.0, 20.0) | 9.0 (7.0, 10.0) | 148.8 (133.0, 160.0) |
|  | p-value | 0.62 | 0.59 | 0.92 | 0.89 | 0.56 | 0.30 | 0.77 | 0.72 |
| Disease recurrence ^┼^ | Yes | 21.0 (17.0, 25.0) | 23.0 (21.0, 24.0) | 19.0 (13.0, 20.0) | 20.0 (19.0, 26.0) | 49.0 (35.0, 57.0) | 16.0 (14.0, 17.0) | 8.0 (3.0, 10.0) | 128.0 (119.7, 142.0) |
|  | No | 24.0 (19.0, 26.0) | 25.0 (22.2, 27.0) | 21.0 (18.0, 23.0) | 23.0 (16.0, 26.0) | 56.0 (49.0, 62.0) | 18.9 (15.5, 20.0) | 8.0 (5.0, 10.0) | 147.3 (126.5, 161.5) |
|  | p-value | 0.24 | **0.13** | 0.24 | 0.81 | **0.17** | **0.19** | 0.63 | **0.18** |

*Spearman’s correlation

┼ Wilcoxon rank-sum test.

Bolded p-values are those with p-value < 0.2.

Supplemental Table 3. Multivariable analysis of clinical and pathologic features associated with better QOL subscale scores.

| Variable |  | ß (95% CI) | | | | | | | | |
| --- | --- | --- | --- | --- | --- | --- | --- | --- | --- | --- |
|  |  | PWB*^┼^ | SWB*^┼^ | EWB*^┼^ | FWB*^┼^ | ECS | Swallowing* | Eating | FACT-E Total*^┼^ |  |
| Age |  | 0.41  (-2.9, 3.7) | 9.53  (-51.5, 70.6) | **51.35**  **(13.0, 89.7)** | -0.25  (-4.1, 3.6) | 0.16  (-0.2, 0.5) | **23.07**  **(1.6, 44.5)** | 0.05  (-0.1, 0.2) | **133.48**  **(36.3, 230.6)** |  |
|  | p-value | 0.80 | 0.76 | **0.01** | 0.90 | 0.32 | **0.04** | 0.34 | **0.01** |  |
| Female Gender |  | -0.41  (-86.1, 85.3) | -1037.81  (-2609.0, 533.3) | -746.68  (-1734.3, 240.9) | -18.76  (-118.9, 81.4) | -3.12  (-11.2, 4.9) | **769.63**  **(217.3, 1322.0)** | -1.74  (-4.3, 0.8) | -1192.36  (-3692.8, 1308.0) |  |
|  | p-value | 0.99 | 0.19 | 0.14 | 0.71 | 0.44 | **0.01** | 0.18 | 0.34 |  |
| Neoadjuvant Therapy |  | 44.90  (-52.1, 141.9) | 1002.41  (-774.9, 2779.7) | -269.77  (-1387.0, 847.4) | -7.54  (-120.9, 105.8) | 2.15  (-7.0, 11.3) | **784.76**  **(160.0, 1409.6)** | 0.23  (-2.7, 3.1) | -12.46  (-2840.9, 2816.0) |  |
|  | p-value | 0.36 | 0.26 | 0.63 | 0.89 | 0.64 | **0.01** | 0.88 | 0.99 |  |
| Clinical TNM Stage III-IV |  | -43.48  (-131.0, 44.0) | -250.74  (-1854.9, 1353.5) | -402.52  (-1410.9, 605.9) | -30.22  (-132.5, 72.1) | -1.95  (-10.2, 6.3) | **-913.87**  **(-1477.8, -349.9)** | -0.13  (-2.7, 2.5) | -1463.25  (-4016.2, 1089.7) |  |
|  | p-value | 0.32 | 0.76 | 0.43 | 0.56 | 0.64 | **<0.01** | 0.92 | 0.26 |  |
| Clavien-Dindo Grade **≥** 2 |  | -36.76  (-101.3, 27.8) | -1016.16  (-2199.2, 166.8) | -538.99  (-1282.6, 204.6) | -55.78  (-131.2, 19.7) | -3.63  (-9.7, 2.4) | 169.64  (-246.2, 585.5) | -1.63  (-3.6, 0.3) | -1568.24  (-3450.9, 314.4) |  |
|  | p-value | 0.26 | 0.09 | 0.15 | 0.14 | 0.24 | 0.42 | 0.09 | 0.10 |  |
| Disease recurrence |  | -111.90  (-238.2, 14.4) | -1399.17  (-3713.9, 915.6) | -399.18  (-1854.2, 1055.9) | -15.13  (-162.7, 132.5) | -9.81  (-21.7, 2.1) | **-905.17**  **(-1718.9, -91.4)** | -2.02  (-5.8, 1.7) | -1484.57  (-5168.4, 2199.3) |  |
|  | p-value | 0.08 | 0.23 | 0.58 | 0.84 | 0.10 | **0.03** | 0.29 | 0.42 |  |

* Box-Cox transformation.

^┼^The residuals not normally distributed after Box-Cox transformation.

Bolded values represent p-value < 0.05.

Supplimental Figure 1

1. Normal emptying (NE) prompt contrast passage from the conduit into the small bowel. B. Delayed emptying (DE) contrast remains in the conduit up to one minute without passage into the small bowel.


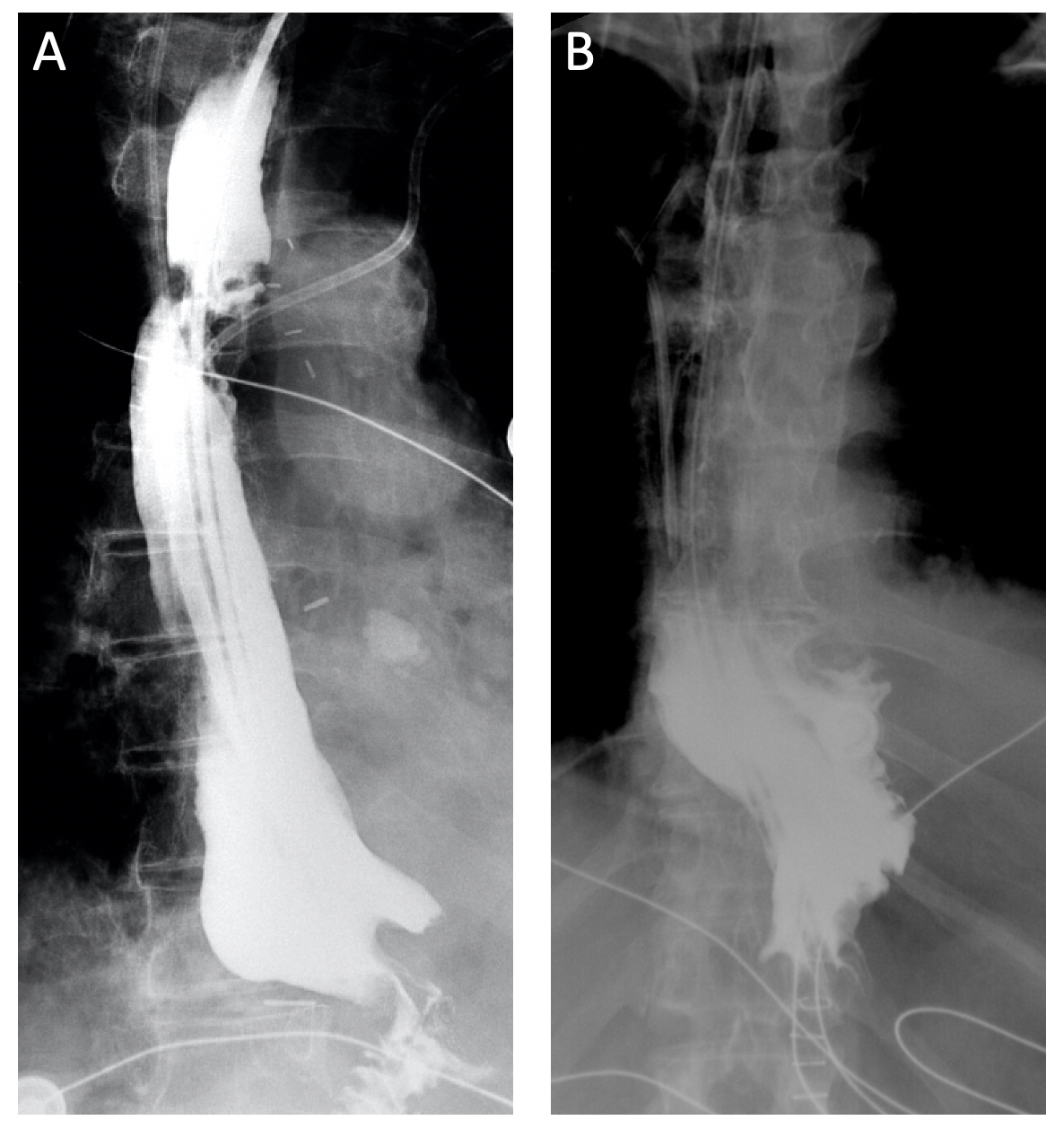

Supplement: Supplementary file 1 [file Datasheet1.docx]
